# vWF correlates with visceral and pericardial adipose tissue in patients with a recent stroke of suspected cardiogenic etiology

**DOI:** 10.1371/journal.pone.0178508

**Published:** 2017-06-01

**Authors:** Antti Tapani Muuronen, Mikko Taina, Juha Onatsu, Miika Korhonen, Kari Pulkki, Pekka Jäkälä, Ritva Vanninen, Pirjo Mustonen

**Affiliations:** 1 Diagnostic Imaging Centre, Department of Clinical Radiology, Kuopio University Hospital, Kuopio, Finland; 2 Institute of Clinical Medicine, Unit of Radiology, University of Eastern Finland, Kuopio, Finland; 3 Department of Medicine, Keski-Suomi Central Hospital, Jyväskylä, Finland; 4 Neuro Center, Kuopio University Hospital, Kuopio, Finland; 5 Department of Clinical Chemistry, University of Eastern Finland, Kuopio, Finland; 6 Eastern Finland Laboratory Centre, Kuopio, Finland; 7 Institute of Clinical Medicine, Unit of Neurology, University of Eastern Finland, Kuopio, Finland; Niigata Daigaku, JAPAN

## Abstract

**Aims:**

A chronically elevated level of von Willebrand factor (vWF) is a common finding in patients with cardiovascular diseases. Obesity is a well-recognized risk factor for thrombotic cardiovascular complications including ischemic stroke, and it has been linked with increased plasma vWF. We evaluated whether elevated plasma levels of vWF associate with areas of visceral (VAT), pericardial (PAT), and subcutaneous adipose tissue (SAT) compartments in patients with acute/subacute stroke.

**Methods and results:**

A total of 69 patients with stroke of suspected cardiogenic etiology were examined. The plasma level of vWF antigen (vWF-ag) was measured both in the acute phase and in the chronic phase three months after stroke. The areas of VAT and/or PAT were assessed with computed tomography. As expected, in stroke patients, the levels of plasma vWF-ag were significantly higher than in the national reference population both in the acute and in the chronic phase. The level of vWF-ag in the chronic phase correlated with the amounts of VAT and PAT, but not with subcutaneous adipose tissue.

**Conclusions:**

These results agree with previous observations of the chronic inflammation/prothrombotic tendency in patients with cerebrovascular disease. Future studies should seek to clarify the role of visceral type adipose tissue in the pathophysiology of ischemic stroke.

## Introduction

Thrombus formation involves several complex reactions and plays an important role in the pathophysiology of ischemic stroke. Von Willebrand factor (vWF) is a multimeric plasma glycoprotein that is a key player in both primary and secondary hemostasis [1]. vWF stabilizes Factor VIII (FVIII) in the circulation, facilitates the adhesion of platelets to the reactive endothelium and subendothelial structures via the glycoprotein (GP) Ib receptor, and subsequent platelet aggregation via the GP IIb/IIIa receptor [2]. In addition to its role in normal haemostasis, vWF is a critical factor in pathological arterial thrombosis (characterized by the presence of high shear forces), and it is also involved in the formation of intracardiac thrombi and venous thromboembolism [3, 4].

Large amounts of vWF can be released from endothelial cells in response to adrenergic stimulation and inflammation and it is a well-known marker of vessel dysfunction in vascular disorders [5]. vWF is an acute phase reactant and therefore high plasma levels are seen in several acute illnesses, including acute myocardial infarction (AMI) and ischemic stroke [6–8]. However, also a persistent elevation of vWF has been reported several months after AMI and stroke, indicative of chronic endothelial activation. A high level of vWF predicts recurrent cardiovascular events, and is also associated with an increased risk of first-ever stroke [9, 10].

Obesity is also a known risk factor for ischemic stroke via a mechanism that remains elusive despite extensive studies. Different adipose tissue depots are associated with different metabolic risks; visceral and pericardial type adipose tissues are considered metabolically more active than subcutaneous adipose tissue. Consequently, increases in the amounts of visceral and pericardial adipose tissues have been associated with the chronic elevation of inflammation markers [11, 12]. According to our previous study, patients with embolic stroke of undetermined source (ESUS) carry increased levels of visceral adipose tissue (VAT) [13]. A positive correlation between VAT and vWF has been reported in obese women, but to the best of our knowledge, no studies in stroke patients have been published [14].

The purpose of this study was to assess the levels of vWF measured three months after an ischemic stroke or transient ischemic attack (TIA) of suspected (but not obvious) cardiogenic or unknown etiology and to evaluate their possible correlations with VAT, pericardial (PAT) and subcutaneous adipose tissue (SAT).

## Methods

The study was approved by the Kuopio University Hospital Research Ethics Board (N:o 82/2004) and all clinical investigations have been conducted according to the principles expressed in the Declaration of Helsinki. Prior to participation in the study, written informed consent was obtained from the patient or the patient's legally authorized representative.

### Patients and study design

**Fig 1:**
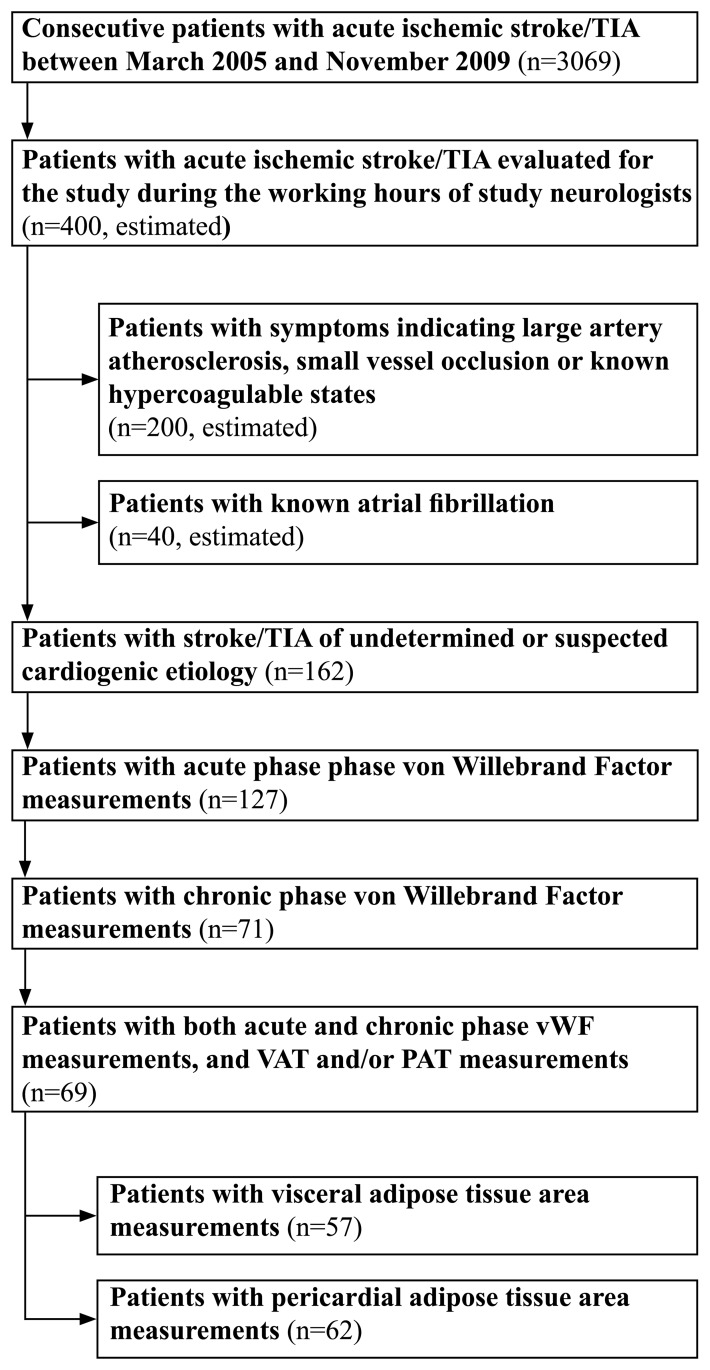
Flow-chart of patient selection.

A flow chart of patient selection is presented in Fig 1. Patients with ischemic stroke or TIA admitted to Kuopio University Hospital between March 2005 and October 2008 were evaluated as candidates for the EmbodeteCT study [13, 15]. A total of 162 patients with stroke/TIA of undetermined or suspected cardioembolic etiology other than atrial fibrillation were recruited by the neurologists involved in the study as a part of their daily clinical work. The suspicion of cardiogenic etiology was based on the characteristic clinical symptoms and/or primary clinical and imaging signs, i.e., simultaneous or sequential infarctions in different arterial territories, hemorrhagic transformation, simultaneous emboli in other organs, reduced consciousness at stroke onset, isolated aphasia, or isolated visual-field defect. Patients with chronic or other previously known atrial fibrillation (AF) were excluded.

According to the study protocol, all patients underwent a comprehensive etiological workup of stroke including computed tomography (CT) angiography of the cervicocranial arteries, ambulatory 24-hour Holter ECG, ECG-synchronized cardiac CT, transthoracic and transesophageal echocardiography, combined with magnetic resonance imaging of the brain and heart when deemed necessary. An additional abdominal CT slice was included to measure VAT. Blood samples were collected at study enrollment and 3 months later. Based on etiological studies, stroke etiology was carefully assessed in each case according to the Trial of Org 10172 in Acute Stroke Treatment (TOAST) criteria ad previously described.

The final study population included 69 patients. Based on the availability of adipose tissue measurements, subjects were analyzed in two groups that overlapped with each other. The first analysis consisted of individuals with vWF-ag and VAT/SAT measurements (n = 57). The second analysis consisted of individuals with vWF-ag and PAT measurements (n = 62).

Subjects were divided into two groups, never-smokers or current/former smokers according to their self-reported smoking status and to have hypertension or diabetes if they were diagnosed either previously or during their hospital stay. BMI was calculated on the basis of height and weight measured from lightly clothed patients without shoes. Based on BMI values, the patients were categorized as normal weight (BMI <25 kg/m^2^), overweight (25 kg/m^2^ ≤ BMI <30 kg/m^2^) or obese (BMI ≥30 kg/m^2^). The fasting total cholesterol, high density lipoprotein (HDL)-cholesterol, low density lipoprotein (LDL)-cholesterol and triglyceride measurements were obtained on the next morning after hospital admission. Body surface area (BSA) was calculated using Mosteller’s formula [16]. The volume of the infarcted cerebral tissue was quantified with CT approximately 24 hours after stroke onset.

### Quantification of the adipose tissue areas

Areas of VAT and PAT were measured as previously described [13, 17]. The area of SAT was measured with computed tomography (16- or 64-slice, 120 kV, 190 mAs, slice thickness 10mm) using an attenuation range from -30 to -190 HU as the area of adipose tissue at the level of the fourth lumbar vertebra excluding VAT [18].

### Measurement of von Willebrand factor antigen in plasma

Blood samples for plasma vWF antigen (vWF-ag) were collected in 3.2% sodium citrate (1) at study enrollment (during acute phase within 72 hours after hospital admission) and (2) 3 months later (chronic phase). Plasma was separated by centrifugation at 2500 g for 15 minutes and stored at -70°C. The vWF antigen levels were analyzed in a specialized national reference laboratory (Finnish Red Cross Blood Service, Laboratory of Haemostasis) within 12 months after sample collection using STA-Liatest vWF (Diagnostica Stago, Asnieres sur Seine, France). All the analyses were performed in duplicates. The reference values for vWF-ag are based on analyses performed on 82 healthy 100% Caucasian individuals in the same national reference laboratory (94.28±29.96%, 95% confidence interval 87.8–100.76%).

### Statistical analysis

Pearson’s correlation coefficient was used to study correlations between chronic phase levels of vWF-ag and VAT, PAT, SAT, BMI, and BSA. Chi-Square was used to study correlations between categorical variables. Continuous variables are expressed as mean ± one standard deviation. In comparative analyses, age adjusted values were used. Categorical variables are presented as absolute values and percentages of the study population. p≤0.05 was considered statistically significant. SPSS 19.0 (1989–2010, SPSS Inc, Chicago, USA) and R version 2.14.0 (2011, The R Foundation for Statistical Computing) software were used for the statistical analyses.

## Results

The clinical characteristics of the study patients are presented in Table 1. The majority of patients were men (66.7%), the mean age of all patients was 61±11 years, and the number of patients with diabetes was low in both groups.

**Table 1:** Patient characteristics and distribution of stroke subtypes based on etiological studies performed on hospital admission.

	Patients with vWF-ag and VAT measurements (n = 57)			Patients with vWF-ag and PAT measurements (n = 62)		
	Men (n = 39)	Women (n = 18)	P	Men (n = 39)	Women (n = 23)	P
Age (years)	59.9±12.1	63.1±9.6	ns.	60.9±12.0	63.4±10.2	ns.
Non-hispanic white, n (%)	39 (100.0%)	18 (100.0%)	ns.	39 (100.0%)	23 (100.0%)	ns.
Paroxysmal atrial fibrillation, n (%)	3 (7.7%)	7 (38.9%)	0.005	3 (7.7%)	7 (30.4%)	0.019
BMI (kg/m^2^)	27.0±3.9	27.5±4.6	ns.	27.3±3.9	27.8±4.4	ns.
Body surface area (m^2^)	2.02±0.18	1.78±0.17	<0.001	2.03±0.18	1.80±0.16	<0.001
Total cholesterol (mg/dl)	170.1±44.4	189.5±31.6	ns.	170.1±42.4	189.5±31.7	ns.
HDL-cholesterol (mg/dl)	38.7±11.7	61.9±18.6	<0.001	38.7±12.8	61.9±17.7	<0.001
LDL-cholesterol (mg/dl)	116.0±39.8	123.7±28.8	ns.	112.1±38.3	116.0±29.2	ns.
Triglycerides (mg/dl)	106.3±79.2	88.6±38.9	ns.	115.1±80.1	88.5±35.9	ns.
Diabetes mellitus, n (%)	1 (3%)	0 (0%)	ns.	2 (5%)	0 (0%)	ns.
Hypertension, n (%)	21 (54%)	10 (56%)	ns.	19 (49%)	12 (52%)	ns.
Current or former smoker, n (%)	9 (23%)	2 (11%)	ns.	8 (21%)	2 (9%)	ns.
Embolic stroke of undetermined source (ESUS)	24 (61.5%)	9 (50.0%)	ns.	27 (69.2%)	14 (60.9%)	ns.
Large artery atherosclerosis,	2 (5.1%)	1 (5.6%)		2 (5.1%)	1 (4.3%)	
Cardioembolism	3 (7.7%)	7 (38.9%)		3 (7.7%)	7 (30.4%)	
Findings supporting both large artery atherosclerosis and cardioembolism	6 (15.4%)	0 (0.0%)		5 (12.8%)	0 (0.0%)	
Small vessel disease	2 (5.1%)	1 (5.6%)		2 (5.1%)	1 (4.3%)	
Technically unsuccessful	2 (5.1%)	0 (0.0%)		0 (0.0%)	0 (0.0%)	

vWF = von Willebrand factor, VAT = visceral adipose tissue, PAT = pericardial adipose tissue, BMI = body-mass index, H/LDL = high-/low-density lipoprotein, ESUS = embolic stroke of undetermined source; ns. = not significant (p>0.05)

The mean values of vWF-ag and the adipose tissue areas are presented in Table 2. Both in the acute and chronic phases, the levels of vWF-ag were considerably higher compared to the reference values obtained from a healthy Finnish population (p<0.0001). In the acute phase, the vWF-ag levels tended to be slightly higher than during the chronic phase. Within the study population there was no statistically significant difference in the vWF-ag levels between normal weight and overweight patients (Table 3).

**Table 2:** Adipose tissue area and von Willebrand factor antigen results.

	Patients with vWF-ag and VAT measurements			Patients with vWF-ag and PAT measurements		
	Men (n = 39)	Women (n = 18)	P	Men (n = 39)	Women (n = 23)	P
VAT area (cm^2^)	210.1±92.0	134.3±54.6	0.002	*NA*	*NA*	0.003
PAT area (cm^2^)	*NA*	*NA*	0.029	14.5±7.6	10.1±6.0	0.021
SAT area (cm^2^)	226.8±91.8	329.3±111.9	0.001	*NA*	*NA*	0.005
vWF-ag, *acute phase* (%)	161.4±65.2	160.0±57.5	ns.	178.8±77.5	160.2±61.5	ns.
vWF-ag, *3 months after stroke* (%)	148.0±47.9	165.7±72.4	ns.	161.9±63.3	161.2±67.4	ns.

ns. = not significant (p>0.05)

**Table 3:** von Willebrand factor antigen results in normal weight and overweight study patients.

	Normal weight (n = 34)	Overweight (n = 35)	P
vWF-ag (%)	166±78	153±45	ns.

ns. = not significant (p>0.05)

**Fig 2:**
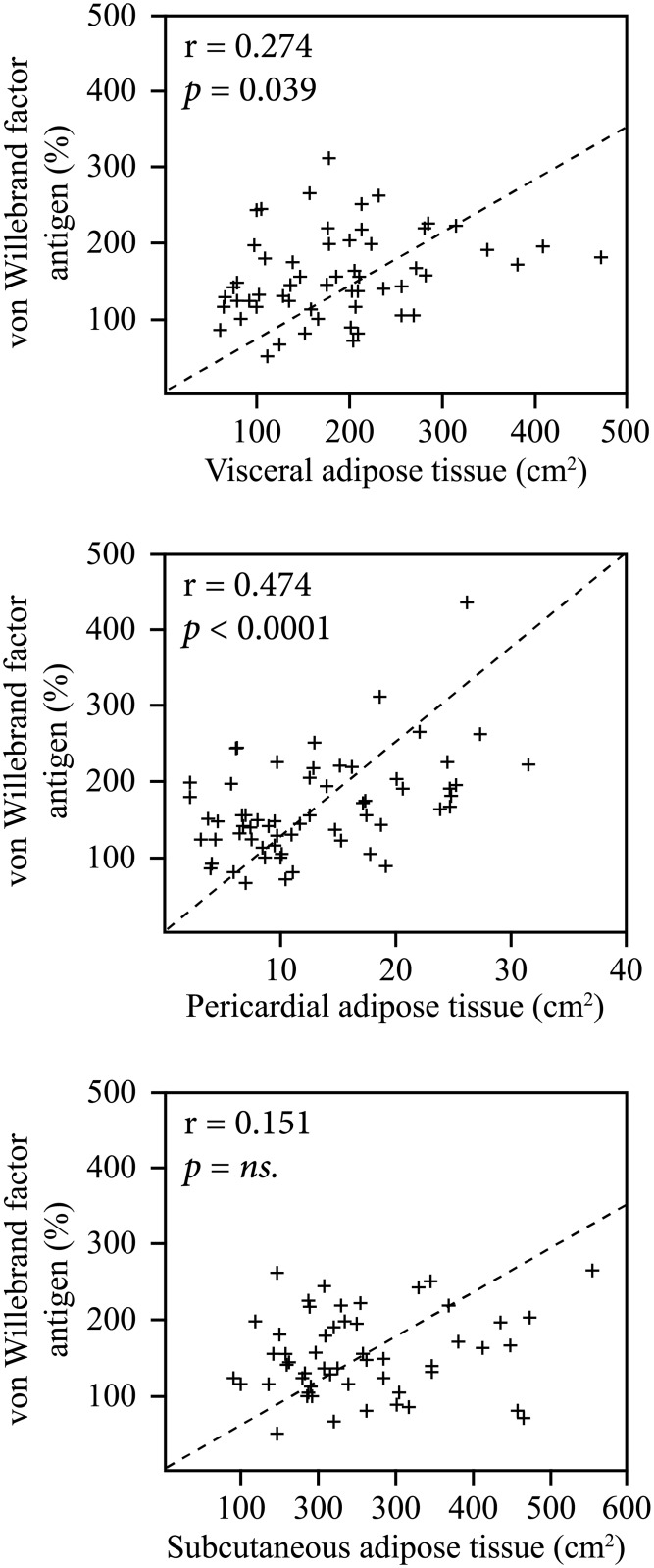
The correlations between chronic phase von Willebrand factor antigen and visceral (n = 57), pericardial (n = 62) and subcutaneous adipose tissue (n = 57).

**Fig 3:**
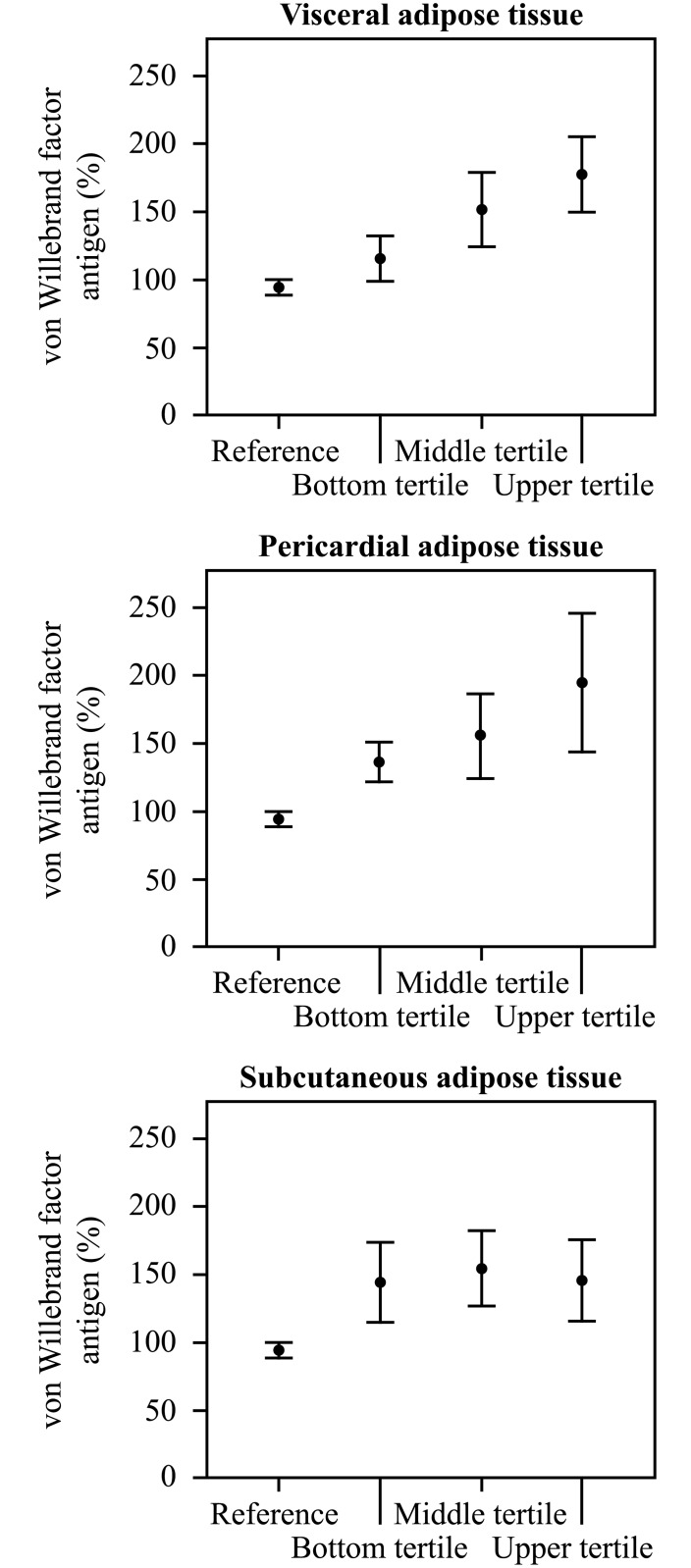
The mean values and their 95% confidence intervals of von Willebrand factor antigen in male patients divided into three groups (n = 13 in each) according to tertiles (bottom, middle and upper tertile) of visceral, pericardial and subcutaneous adipose tissue in comparison to the national reference values.

Fig 2 shows the correlations between chronic phase (3 months) vWF concentrations and the amount of specific adipose tissue depots. In the whole study group, the level of chronic phase vWF-ag correlated significantly with the areas of VAT (r = 0.274, p = 0.039) and PAT (r = 0.474, p<0.0001), but not with SAT, BMI or BSA. The correlation between VAT and vWF-ag was significant in males (n = 39, r = 0.458, p = 0.003) and in the subgroup of male patients with ESUS (n = 24, r = 0.450, p = 0.027). The number of female patients was small but the correlations showed similar trends (data not shown). Fig 3 shows the vWF-ag levels in male patients divided into three groups (n = 13 in each) according to tertiles of VAT, PAT, and SAT as compared to the reference values.

The number of patients within categories of stroke etiology according to TOAST criteria is shown in Table 1. Over half of the patients had ESUS. There were no statistically significant differences in vWF-ag levels, VAT, PAT, or SAT between the etiological subgroups. Ten patients (14.5%) were diagnosed with paroxysmal AF in an ambulatory 24-hour Holter ECG study performed as a part of the etiological studies being conducted after stroke. Patients with paroxysmal AF had higher levels of vWF-ag than others at three months after stroke (205.1± % vs. 142.6± %, p = 0.023). Adipose tissue areas or the level of vWF-ag were not associated with volumes of infarcted brain tissue.

## Discussion

We studied the plasma levels of vWF antigen and their correlation with the amount of adipose tissue in different locations in patients with ischemic stroke of suspected cardiogenic origin or ESUS. The main result of our study was that in patients with a history of a recent (3 months earlier) stroke, the level of vWF-ag correlated with the areas of visceral and pericardial adipose tissue, but not with its subcutaneous counterpart or BMI. We also found that the level of vWF-ag remained chronically elevated in stroke patients.

The relationship of VAT to vWF has not been previously investigated in stroke patients. However, many studies have described an association between obesity and stroke. Obesity has been linked with multiple secondary conditions (diabetes, hypertension, dyslipidemia), which are known risk factors for ischemic stroke [19–21]. Furthermore, the amount and location of adipose tissue are associated with variable risks of cardiovascular diseases [22]. Previous studies have shown that VAT and PAT are correlated with each other and also linked with cardiovascular diseases, diabetes mellitus, hypertension, and dyslipidemia [23, 24]. It has been postulated that low-grade systemic inflammation plays a role in the obesity-associated increased risk of cardiovascular diseases. Adipose tissue acts as a major endocrine organ. Adipocytes and other cells in adipose tissue secrete a wide range of hormones and cytokines including interleukins 1 and 6 and plasminogen activator inhibitor-1 (PAI-1) [25]. Visceral adipose tissue secretes higher levels of inflammatory factors than subcutaneous adipose tissue [26]. Accordingly, an excess ectopic adipose tissue is associated with increased systemic low-grade inflammation whereas a similar association has not been established with subcutaneous adipose tissue.

Plasma levels of vWF are determined by genetic factors (ABO blood groups and vWF mutations) and by non-genetic factors (i.e. aging, impaired nitric oxide production, inflammation, free radical production and diabetes) [27–29]. Moreover, the level of vWF can be increased either chronically or transiently due to an acute condition. In addition to being chronically elevated in patients with cerebrovascular disease, the plasma level of vWF is known to increase in acute ischemic stroke [7]. In the course of acute myocardial infarction with ST-elevation, the levels of vWF are elevated at 24 hours, peak at 48 to 72 hours, before returning to baseline at around day 14 [6]. Previous studies have shown that the high level of vWF may be used to identify acute stroke patients with an increased risk of death [8]. Moreover, an early rise of vWF predicts an adverse outcome in patients with unstable coronary artery disease [30].

Several studies have linked chronically increased plasma concentrations of vWF to an increased risk for thrombotic complications, including acute coronary syndrome and ischemic stroke. While in the general population, the association between vWF and thromboembolic cardiovascular events is weak, in patients with a pre-existing cardiovascular disease, the level of vWF can be used to predict adverse cardiovascular events [31]. An elevated level of vWF predicts the appearance of the first stroke, recurrent myocardial infarction and death in patients with coronary artery disease [9, 32, 33]. In one case-control study of 600 stroke patients, chronically increased levels of vWF measured three months after ischemic stroke were associated with cardioembolic or cryptogenic etiology of stroke [7]. In addition, patients with permanent or paroxysmal AF exhibited elevated levels of vWF.

Our results of the elevated level of stable phase vWF in a stroke patients are in line with previous observations of the chronic inflammation/pro-thrombotic tendency in patients with cerebrovascular disease. The correlations found between VAT, PAT, and vWF may reflect increased inflammatory or pro-thrombotic activity in these adipose tissues whereas SAT and general obesity assessed by BMI did not display a similar correlation. Our study population consists mainly of stroke patients without the most obvious stroke etiologies (LAA and FA). This selected stroke population includes also patients with ESUS, i.e. cases where the stroke remains unclear despite extensice studies [34]. In this population further studies are needed to clarify the pathomechanics behind. We hypothesize that in this kind of stroke population the visceral type adipose tissue, by promoting a pro-coagulative state (in this study assessed as the increased level of vWF-ag), could play a role in the pathophysiology of stroke.

The current study has some limitations. Due to the strict patient selection, our results cannot be generalized to a general stroke population. However, we have a well-characterized population of stroke patients with suspected cardiogenic etiology, and even more specifically, a well-characterized subpopulation with ESUS. The second limitation is the relatively small number of patients evaluated, especially in the subgroups.

Our study reveals that chronic phase vWF-ag levels correlate with the amounts of visceral and pericardial type adipose tissue in patients with recent ischemic stroke of suspected cardiogenic origin. In a subpopulation of male patients with ESUS, the correlations were even stronger, providing valuable information on the association between adipose tissue distribution and chronic inflammation and prothrombotic activity in ESUS. Prospective studies with a larger sample size will be needed to clarify the importance of our findings in the future.

## Supporting information

S1 Table Correlations between adipose tissue compartments and chronic phase inflammation markers. (DOCX)
