# Supplementary material for: vWF correlates with visceral and pericardial adipose tissue in patients with a recent stroke of suspected cardiogenic etiology
Source: PLoS One. 2017 Jun 1;12(6):e0178508. doi: 10.1371/journal.pone.0178508 (PMC5453529; doi:10.1371/journal.pone.0178508)
Supplement: S1 Table — (DOCX) [file pone.0178508.s001.docx]

**S1 Table.** Correlations between adipose tissue compartments and chronic phase inflammation markers.

|  | **IL-1RA** | **IL-1β** | **IL-6** | **IL-10** | **IL-18** | **TNFα** | **CRP** |
| --- | --- | --- | --- | --- | --- | --- | --- |
| **Visceral adipose tissue** | r=0.068  p=0.624 | r=0.118  p=0.397 | r=0.202  p=0.143 | r=-0.044  p=0.753 | p=-0.120  p=0.388 | r=-0.027  p=0.846 | r=-0.036  p=0.798 |
| **Pericardial adipose tissue** | r=-0.066  p=0.615 | r=-0.048  p=0.714 | **r=0.304**  **p=0.018*** | r=-0.175  p=0.181 | r=0.118  p=0.371 | r=0.045  p=0.736 | r=0.029  p=0.826 |
| **Subcutaneous adipose tissue** | r=0.038  p=0.791 | r=0.029  p=0.841 | r=0.181  p=0.209 | r=-0.208  p=0.147 | r=-0.018  p=0.902 | r=0.024  p=0.868 | r=0.041  p=0.778 |
